# Strain-Based Assessment to Evaluate Damage Caused by Deep Rolling

**DOI:** 10.3390/ma17092163

**Published:** 2024-05-06

**Authors:** Tobias Pertoll, Martin Leitner, Christian Buzzi, László Boronkai

**Affiliations:** 1 Institute of Structural Durability and Railway Technology, Graz University of Technology, Inffeldgasse 25/D, 8010 Graz, Austria; 2 Siemens Mobility Austria GmbH, Eggenberger Strasse 31, 8020 Graz, Austria

**Keywords:** strain-based damage assessment, mechanical surface treatment, deep rolling, railway axle, 34CrNiMo6

## Abstract

The positive effects of deep rolling on fatigue strength—reduced surface roughness, work hardening and compressive residual stress—in the near-surface region are achieved by controlled high plasticisation of the treated material. However, excessive and/or repeated plasticising poses a risk of damage to the machined component. This paper investigates the damage caused by deep rolling of a railway axle. Two sections of the axle are experimentally deep rolled repeatedly at different feed rates until damage is detected. For comparative analysis, these experiments are numerically analysed and the damage is assessed using the strain-based damage calculation. The results are compared and a damage sum of ~120% is evaluated for both tests, thus developing a reliable and conservative assessment method. The single deep rolling treatment at a feed rate of 0.25 mm causes damage of 6.1%, and at a feed rate of 0.5 mm, damage of 4.7%. The developed and experimentally validated evaluation method allows for investigating the limits of applicability of different deep rolling parameters. The influence of the deep rolling force and feed rate and a proposed optimisation with multiple deep rolling with reduced deep rolling forces are investigated.

## 1. Introduction

Deep rolling is used to improve fatigue strength and inhibit crack initiation and propagation. The post-treatment process is most commonly used on rotationally symmetric components. During this process, a deep rolling tool is pressed against the rotating surface of the component to be machined and simultaneously moved in a longitudinal direction at a constant feed rate, very similar to the turning process. However, no material is removed in the contact between the tool and the component; instead, plastic deformation is induced. This reduces surface roughness, work-hardens the area near the surface and introduces residual compressive stresses [1,2]. Three effects to increase fatigue strength are achieved in one operation through controlled plasticising. The process can be easily integrated into the manufacturing process as a final operation and is therefore comparably cost effective. As a result, deep rolling is widely used in a variety of industries, for example, in the automotive industry for the post-treatment of crankshafts, in the aerospace industry for the post-treatment of turbine components, and in the rail vehicle industry for the post-treatment of railway axles [1,3,4,5,6,7].

Railway axles, which are the primary focus of this study, are the connecting part with the mounted wheels between the superstructure and the railway vehicle. The railway axles are designed to transmit the track guiding forces, the tractive and braking forces and the bending load caused by the vehicle weight. The main load is the rotating bending load. These components are highly relevant to safety, and their failure can lead to train derailment and the associated risk to passengers. However, research and engineering work is underway to further develop railway axles. The aim is to reduce axle mass and thus unsprung mass. This will reduce the dynamic forces in wheel–rail contact, thereby reducing wear and optimising energy consumption during operation. This unsprung mass of the rolling stock is also decisive for the calculation of track access charges [8,9].

Currently, the benefits of deep rolling are not explicitly emphasised in the European standards EN 13103-1 [10] and EN 13261 [11] for the design of railway axles. However, the integration of these benefits remains critical to achieving a long-term mass reduction in the new manufactured components.

In high-strength steels, such as the 34CrNiMo6 steel investigated in this study, it is the compressive residual stresses introduced that make the greatest contribution to improving fatigue and crack propagation behaviour [2,12,13]. These residual stresses and near-surface work hardening are achieved by plasticising the material. The increased work hardening is achieved by increasing the dislocation density in the material. These changing properties are analysed in terms of residual stress measurements and FWHM evaluation on the same railway axles used for the tests here, and the results are presented in [14].

In addition to the measurements, a numerical deep rolling simulation model is presented and validated with the residual stress measurements. The simulation model is used to investigate the influence of deep rolling parameters and optimisations on the residual stresses applied, and the results are presented in [15]. The influence of these on cracking behaviour [16] and fatigue strength [17] is also investigated. The application of the highest deep rolling force shows the most significant depth effect on the residual stresses applied, the best crack propagation behaviour, and also the best result in terms of fatigue strength. This would lead to the idea that better deep rolling results can be achieved by further increasing the deep rolling force. However, this is not the case; as mentioned above, plasticising changes the material and increases the dislocation density. This is further increased by increasing forces or repeated deep rolling, and these can coalesce and cause microcracking [18,19,20,21]. This, in turn, reduces the service life of the component in service. This phenomenon is often reported for the shot peening process, referred to as “over peening” [22,23,24,25,26,27]. With increasing Almen intensity [28,29,30], fatigue strength increases, reaches an optimum, and then decreases again, leading to suboptimal results. Similar behaviour is assumed for deep rolling, but no literature on this could be found.

This is precisely where the present study comes in as it investigates this behaviour. It is assumed that each deep rolling overrun with the appropriate deep rolling parameters causes “partial damage”. However, up to a certain point this has a positive effect on fatigue strength, which has been mentioned several times in the literature [31,32,33,34], and then the achievable benefit diminishes.

In this paper, the damage introduced is investigated experimentally and computationally. Experimental deep rolling is carried out repeatedly with the same parameters until damage is found in the form of surface cracks. Magnetic particle inspection is used for verification, and tests are carried out on two railway axle parts with two different deep rolling parameters. This repeated deep rolling is used to determine the limits of damage caused by the process application and to validate the calculation methodology.

Damage evaluation is performed using a strain-based damage assessment approach. On the one hand, this approach is based on allowable material values, in this case a strain curve estimated according to the Uniform Material Law (UML) [35,36] and checked with test results. On the other hand, the strain caused by deep rolling has to be determined. The strain loads are determined using the numerical deep rolling simulation model. The time history of strain and stress over time is analysed and subdivided for each processing step to determine the strain amplitudes that occur. Partial damage is determined according to the strain-based damage approach considering the influence of mean stress after [37]. The “partial damage” is summarised to obtain the damage of a deep rolling overrun according to the linear damage accumulation, the Miner rule [38].

The deep rolling application is also repeated in the simulation, the damage is determined and compared with the test result. The comparison is promising, a valid damage sum is determined, and thus a conservative calculation method can be established to determine the damage caused by deep rolling.

This approach allows an evaluation of the influence of deep rolling parameters and optimisations on the applied damage. The influence of the most influential deep rolling parameters, the deep rolling force and the feed rate, and the optimisation with multiple deep rolling with reduced deep rolling forces are investigated.

The scientific contribution of the article can be summarised as follows:

- Experimental determination of the number of deep rolling overruns until a certain surface damage is detected.
- Development of a calculation method to determine the damage caused by deep rolling based on strain-based damage assessment and the linear damage accumulation.
- Validation of the calculation method using the results of the experiments.
- Determination of the impact of the deep rolling force, feed rate and deep rolling optimisation using multiple deep rolling with reducing forces on the damage introduced.

## 2. Materials and Methods

Deep rolling is used to increase the fatigue strength of railway axles. The increase in service life is achieved by controlled plasticisation of the near surface area. This process involves the reduction of surface roughness, work hardening of the near surface area and the introduction of compressive residual stresses. The increase in fatigue strength achievable depends on the correct choice of parameters. Similar to over-peening in shot peening, it is assumed that over-deep-rolling, and thus a deterioration in component properties, can occur due to excessive plasticising. This means that the material is subjected to high loads, resulting in high local strains well into the plastic range. In deep rolling, such undesirable effects may arise from excessive forces, too low feed rates, or repeated deep rolling of the same area.

The aim of this investigation is to develop a reliable calculation method that can accurately assess the damage caused by deep rolling. The calculation is based on a numerical simulation model of the deep rolling process combined with a strain-based damage assessment. The result of the calculation will be compared with the result of the experimental investigation. Two experiments are performed for validation. The parameters used in the experiment and the simulation are chosen identically and are summarised in Table 1.

The difference between the two experiments is the feed rate. With different feed rates, the same area is processed a different number of times and therefore plasticised differently. The feed rate *FR*_1_ of 0.25 mm is used in Experiment 1, and the feed rate *FR*_2_ of 0.5 mm is used in Experiment 2; all other parameters remain the same. When the term “feed rate” is used in this publication, it always refers to the actual distance between the tools on the component. The experiments are carried out using a double roller tool with opposed discs. Due to the helical machining, half the feed rate of the lathe tool carriage feed is applied to the component surface. Therefore, for a feed rate *FR*_1_ = 0.25 mm on the component, a feed rate of 0.5 mm is set on the machine, and for a feed rate *FR*_2_ = 0.5 mm on the component, a feed rate of 1.0 mm is set on the lathe.

### 2.1. Experimental Work

**Figure 1:**
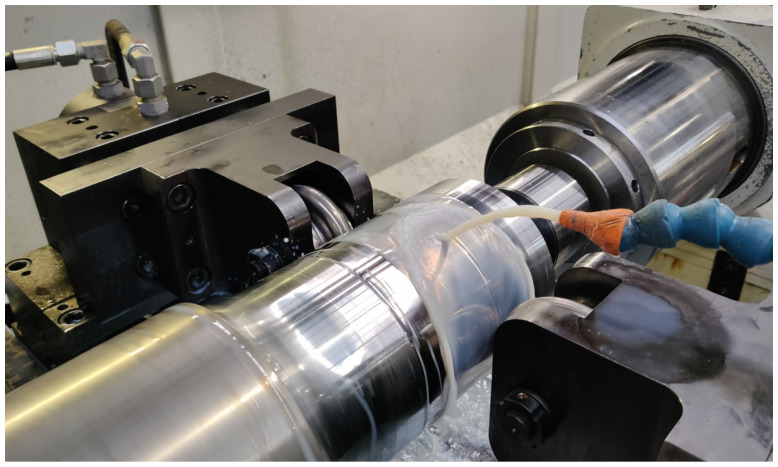
Application of the deep rolling process during experiments.

Experiments 1 and 2 are carried out on two cylindrical sections of a railway axle, which has been used in previous studies [14]. As mentioned above, all the deep rolling parameters remain identical, except for the feed rate. The deep rolling tool is mounted on a lathe on the tool carriage, and the railway axle parts are clamped. The test set-up is shown in Figure 1. A twin tool with opposing discs is used for deep rolling. The dimensions of the discs are given in Table 1. The deep rolling force *F_DR_* of 20 kN is generated hydraulically. The force is increased with a ramp at the beginning of the machining process, then held constant for 96 mm in the test area and reduced again at the end. A rotational speed of 180 rpm is used. During machining, the process is cooled and lubricated with coolant.

For the investigation, the deep rolling process described for Experiments 1 and 2 is repeated in the specified areas with identical parameters until damage is found on the railway axle. Magnetic particle inspection is used to detect the damage. The surface is repeatedly inspected for cracks between overruns. The purpose of the test is to determine the number of deep rolling overruns until damage occurs. For validation, this result is compared with the results of the strain-based damage assessment. The calculation methodology is presented in the following section.

### 2.2. Strain-Based Damage Assessment

In order to optimise the application of the deep rolling process, it is important to calculate the damage caused by the treatment itself. An approach based on strain-based damage assessment is used. The permissible strength of the material is determined using the strain life curve according to the Uniform Material Law (UML) [35] and extended by the influence of mean strain/stress using the Smith, Watson & Topper (SWT) [37] approach. The strain amplitudes occurring during deep rolling are analysed by a simulation model of the deep rolling process, and the partial damage introduced by each individual tool pass is determined. The total damage that occurs in a deep rolling overrun can be calculated from the partial damage using the linear damage accumulation approach. The calculation methodology is explained in detail in the following subsections.

#### 2.2.1. Strain-Life Curve

The cyclic strain strength of the railway axle material is determined using UML. The UML determines the strain-life curve based on the Young’s modulus and the ultimate tensile strength of the material. Its equation is based on the approach of [35], is also presented in [36] and is given by Equation (1). The relationship between strain amplitude *ε_a_* and the tolerable number of load cycles *N* is given. The equation is composed of an elastic *ε_a,e_* and a plastic *ε_a,p_* part. The other parameters used are defined in Table 2.

(1) $$\varepsilon_{a}=\varepsilon_{a,e}+\varepsilon_{a,p}=\left(\frac{{\sigma \prime }_{f}}{E}\right)\times {\left(2N\right)}^{b}+{\varepsilon \prime }_{f}\times {\left(2N\right)}^{c}$$

The Young’s modulus and the tensile strength for the 34CrNiMo6 steel investigated are taken from the tensile test results in [14]. The tests are carried out on the same railway axle as used for the experiments presented in this study. Table 2 summarises the numerical values used and the parameters calculated for the UML definition.

**Figure 2:**
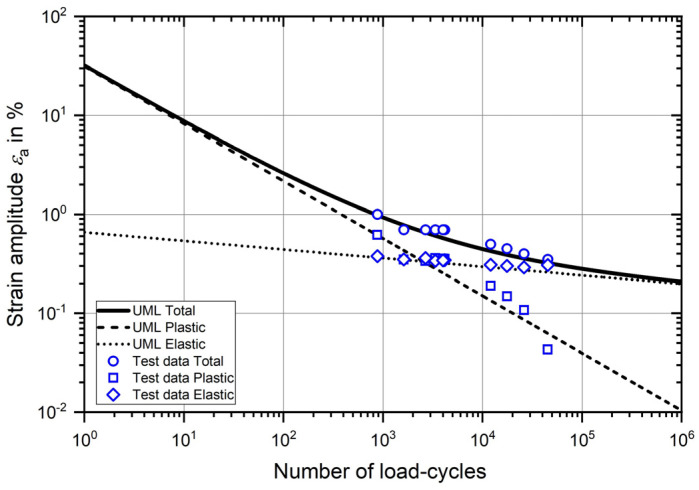
Strain-life curve according to UML and cyclic test results.

Figure 2 shows the strain-life curve according to the UML using the parameters specified above in Table 2. The total strain-life curve and also its elastic and plastic components are shown. The validity of the UML for the material analysed is verified by the test results. In addition to the tensile tests, numerous cyclic tests were carried out on specimens taken from the analysed railway axle, some of which are presented in [14]. The results which include different applied strain amplitudes and a constant load strain ratio of R_ε_ = −1 are also plotted in the same diagram. Again, the total strain amplitude and the elastic and plastic components are plotted. The estimated strain-life curve shows good agreement with the test results.

#### 2.2.2. Mean Stress/Strain Consideration—SWT Approach

The UML and the resulting strain-life curve are valid for an R-ratio of *R* = −1, and therefore, no mean stress/strain is considered. The repeated side-by-side rolling of the deep rolling disc tools, offset by the feed rate during deep rolling, causes mean stress/strain, and therefore, different conditions prevail for each rolling pass. A common approach to considering mean stress/strains is the SWT approach [36,37]. The damage parameter *P_SWT_* is calculated according to the following equations. The same parameters are used as in the UML and are given in the defined Table 2. The additional parameter *σ_max_* considers the influence of the mean stress/strain.

(2) $$P_{SWT,i}=\sqrt{\sigma_{max,i}\times \varepsilon_{a,i}\times E}$$

(3) $$P_{SWT,i}=\sqrt{{\sigma \prime }_{f}^{2}\times {\left(2N_{i}\right)}^{2b}+{\sigma \prime }_{f}\times {\varepsilon \prime }_{f}\times E\times {\left(2N_{i}\right)}^{\left(b+c\right)}}$$

Equations (2) and (3) can be set equal, resulting in a mean stress/strain-dependent relationship between strain amplitude *ε_a,i_* and tolerable number of load cycles *N*. As the mean stress/strain changes for each machining of each deep rolling tool, the damage parameters are recalculated for each machining. This is described by the indices *i* in the equation for the dependent equation parameters. All other parameters in the equation are constant.

#### 2.2.3. Numerical Simulation Model of the Deep Rolling Process

The deep rolling simulation model presented in [14] and set up in MSC Marc is used to determine the strain amplitudes. To determine the damage caused by deep rolling, the allowable number of load cycles *N* is determined for each deep rolling disc tool based on the strain amplitude that occurs. The simulation model validated with residual stress measurements is used to analyse the influence of deep rolling parameters on the introduced residual stresses [15]. The simulation model basically consists of a simplified cuboid section of the railway axle and deep rolling disc tools. An elastic-plastic Chaboche material model is applied to the railway axle section. The material model is parameterised on the basis of the uniaxial cyclic tests mentioned in Section 2.2.1 and presented again in [14]. The numerical model is capable of simulating the same parameters as used in the experiment. The difference from real deep rolling is in the movement of the discs rather than the railway axle within the simulation. The cuboid section of the railway axle is constrained with symmetry constraints on all sides except the surface to represent the surrounding material. Several discs are modelled in parallel, aligning in dimensions with those used in the experimental setup. These roll over the surface one after the other. First, the deep rolling force of 20 kN is applied, and frictional contact (*µ* = 0.1) causes the disc to roll over the surface while the force is kept constant. Finally, the disc stops and the force is reduced again. This process is repeated for each disc in turn to reduce simulation time. The distance between the discs is chosen so that they do not influence each other and do not affect the simulation result.

**Figure 3:**
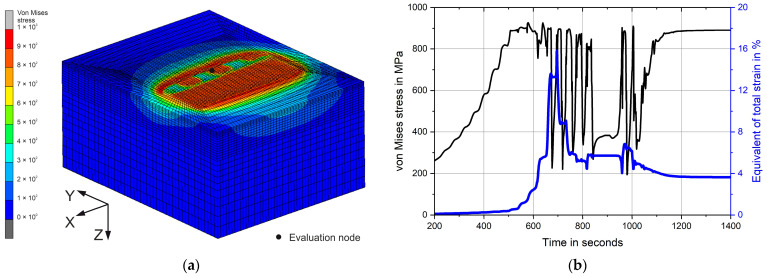
Simplified railway axle section of the simulation model during simulation procedure of Experiment 2 with marked evaluation node (**a**) and von Mises stress and equivalent of total strain over simulation time evaluated there (**b**).

Figure 3a demonstrates the railway axle section during deep rolling. The von Mises stresses are presented. Small areas of high local stresses, further along the *Y*-axis, represent a contact between the disc tool and the simulation model. The larger area of high stress, closer to the *Y*-axis, shows the residual stresses already introduced. To determine the stresses and strains that occur during deep rolling, a history plot is evaluated from the evaluation node shown in the figure on the surface in the centre of the simulation model. The evaluation of the evolution of the von Mises stress and the equivalent of total strain over the duration of the simulation is shown in Figure 3b. For better visualisation, the result of a deep rolling pass from Experiment 2 with a higher feed rate is shown as an example.

#### 2.2.4. Strain Amplitude Definition

**Figure 4:**
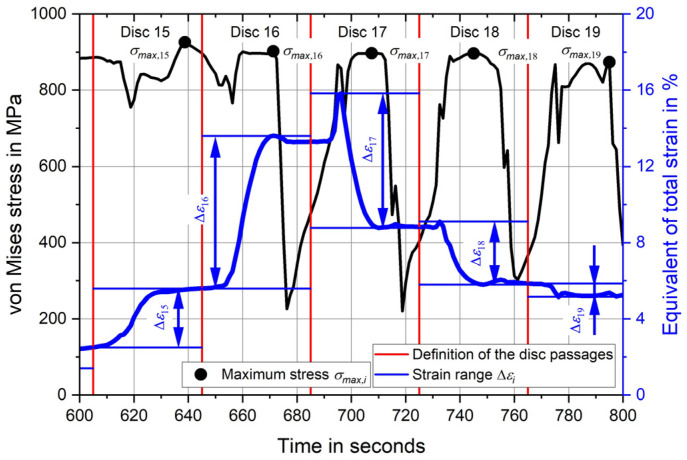
Definition of the strain range and maximum stress for each disc.

From the stress and strain over time curves shown in Figure 3b, the required strain changes and maximum stresses for each disc are derived. This is now explained using Figure 4, on which a section of Figure 3b is shown. At the beginning, the time sequence for each disc is subdivided. This is shown by the red lines. In between there are the strain and stress changes for each passing disc. The time intervals with the largest strain changes, discs 15 to 19, are shown.

The stress and strain state during deep rolling is multiaxial and changes with each disc passage. Initially, deep rolling takes place in front of the evaluation point, and then the discs approach the evaluation point at a constant feed rate. This is followed by several deep rolling passes with the discs in direct contact with the evaluation point, and finally the discs move farther and farther apart. In order to be able to compare the strains and stresses with the uniaxial strain-live curve, the equivalent of total strain and the equivalent stress, the von Mises stress, are used for the damage calculation.

To define the strain range for each disc, the maximum strain value *ε_max_* and the minimum strain value *ε_min_* of the comparative strain occurring in the observed time period are determined and the difference is calculated. The corresponding strain amplitude *ε_a,i_* is determined by halving the strain range Δ*ε_i_* according to Equation (4).

(4) $$\varepsilon_{a,i}=\frac{{∆\varepsilon }_{i}}{2}=\frac{\varepsilon_{max,i}-\varepsilon_{min,i}}{2}$$

Finally, the maximum stress *σ_max,i_* is missing. Therefore, the maximum value of the von Mises stress is determined for the time period of the disc passes.

#### 2.2.5. Damage Assessment and Linear Damage Accumulation

Now that all parameters are defined, the allowable number of load cycles *N_i_* can be determined for each deep rolling disc passage *i*. This determination is achieved by solving Equations (2) and (3). Conversion to the strain amplitude *ε_a,i_* gives Equation (5).

(5) $$\varepsilon_{a,i}=\frac{{\sigma \prime }_{f}^{2}\times {\left(2N_{i}\right)}^{2b}+{\sigma \prime }_{f}\times {\varepsilon \prime }_{f}\times E\times {\left(2N_{i}\right)}^{\left(b+c\right)}}{\sigma_{max,i}\times E}$$

This equation is solved using a numerical non-linear system optimisation solver. The allowable number of load cycles *N_i_* for each disc passage *i* is then obtained, which is calculated as a function of the strain amplitude *ε_a,i_* and taking into account the mean stress/strain by *σ_max,i_*.

The allowable number of load cycles *N_i_* is used to calculate the partial damage *D_i_* by Equation (6). This is calculated by dividing the occurring number of load cycles *N* by the allowable number of cycles *N_i_*. However, as the allowable number of cycles varies for each treating disc, no classification is used and therefore the number of load cycles is always set to *N* = 1.

In order to determine the damage of a deep rolling overrun *D_j_* from the partial damage *D_i_* accused by the individual discs, linear damage accumulation is applied, so that the sum of the partial damage of disc 1 (*i* = 1) is calculated up to the total number of discs *i* = *N_D_* required in the simulation.

(6) $$D_{j}=\sum_{i=1}^{N_{D}}D_{i}=\sum_{i=1}^{N_{D}}\frac{N}{N_{i}}$$

### 2.3. Investigated Parameters and Optimisation

The functionality-tested calculation method is designed to evaluate the influence of deep rolling parameters and optimisations on the damage caused. This is to ensure that even with repeated deep rolling or lower feed rates, for example, no damage and thus component failure is caused by the process application itself.

In [15], the influence of deep rolling parameters and an optimization proposal regarding the introduced residual stresses is investigated. The most influential parameters, specifically the deep rolling force and feed rate, and the proposed optimisation are selected and investigated in this study.

The damage calculation is used for the deep rolling forces 20 kN, 15 kN, 10 kN, 5 kN and 2 kN, and the results are presented in Section 3.3.1.

The feed rates 0.25 mm and 0.5 mm are already used for validation in Experiment 1 and Experiment 2. In addition, the investigation is extended to the feed rates 1.0 mm and 2.0 mm. The results are presented in Section 3.3.2.

The optimisation proposal is based on repeated deep rolling with reduced deep rolling forces. The combinations 20 kN followed by 5 kN, 20 kN followed by 10 kN, and 20 kN followed by 10 kN and finally 5 kN are analysed. The results are presented in Section 3.3.3.

## 3. Results and Discussion

This section presents and interprets the results: firstly, the results of the experiments carried out, followed by the results derived from calculations and their comparison with the experiments. Finally, the calculation method is applied to assess the deep rolling parameters, the deep rolling force and the feed rate, and to the optimisation presented, and the influence of these on damage is presented.

### 3.1. Experimental Work

This section presents the results of the experiments described in Section 2.1. Two experiments are carried out on two cylindrical parts of the railway axle. The deep rolling overruns are repeated until damage is detected by magnetic particle inspection. In Experiment 1, with a feed rate *FR*_1_ of 0.25 mm, the first damage is detected after *j* = 23 deep rolling overruns and in Experiment 2, with a feed rate *FR*_2_ of 0.5 mm, after *j* = 48 deep rolling overruns.

In Experiments 1 and 2, an additional two deep rolling overruns were conducted before completion of the tests to further investigate the crack formation after crack initiation is detected.

**Figure 5:**
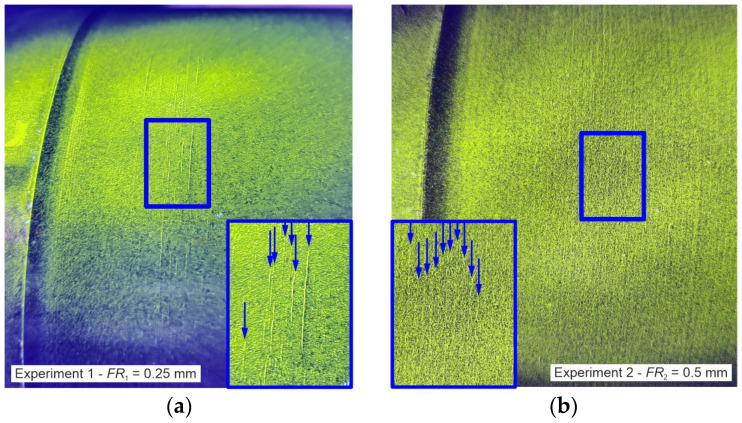
Result of the magnetic particle inspection for Experiment 1 after 25 deep rolling overruns (**a**) and Experiment 2 after 50 deep rolling overruns (**b**).

Figure 5a shows the result of the magnetic particle inspection of Experiment 1 after 25 deep rolling overruns. Fluorescent liquid and ultraviolet light are used to make the cracks visible. In particular, it demonstrates the presence of elongated cracks that are oriented circumferentially around the railway axle. These occur irregularly and cannot be attributed to the feed rate used.

The result of the magnetic particle inspection test of Experiment 2 after 50 deep rolling overruns is shown in Figure 5b. The surface is covered with fine cracks. These occur regularly at the feed distance of the disc tools.

In both experiments with different feed rates it was possible to induce clear damage to the surface, so that the result can be compared with the result of the calculation and the corresponding damage sum can be determined.

### 3.2. Strain-Based Damage Assessment and Comparison with the Experimental Results

The calculation method presented in Section 2.2 is now applied to the first deep rolling overrun of both Experiments 1 and 2. This involves simulating the process at the appropriate feed rate, analysing the strains and stresses, determining the partial damage for each disc passage and calculating the damage for a deep rolling overrun using linear damage accumulation. The damage determined from a deep rolling overrun of Experiment 1 is 6.1%, and the damage for Experiment 2 is 4.7%. The double feed of Experiment 2 causes less damage because the evaluation point, and any point on the surface, is treated less frequently and therefore fewer strain and stress cycles occur.

To validate the assessment method, it must be compared with the experimental results. In order to achieve this, the deep rolling overruns *j* must also be repeated in the simulation. For Experiment 1, six deep rolling overruns can be simulated, and for Experiment 2, 14 overruns can be simulated due to the higher feed rate and therefore shorter simulation time. To simulate this repeated deep rolling with the specified number of overruns, both simulations require a simulation time of over three months and a disc space requirement of over 3 TB.

The strain and stress curves over time are analysed for the deep rolling overruns, and the damage *D_j_* is determined for each deep rolling overrun. These are accumulated, and the total damage *D* is calculated from the first overrun *j* = 1 to the analysed number of deep rolling overruns *j* = *N_O_*.

**Figure 6:**
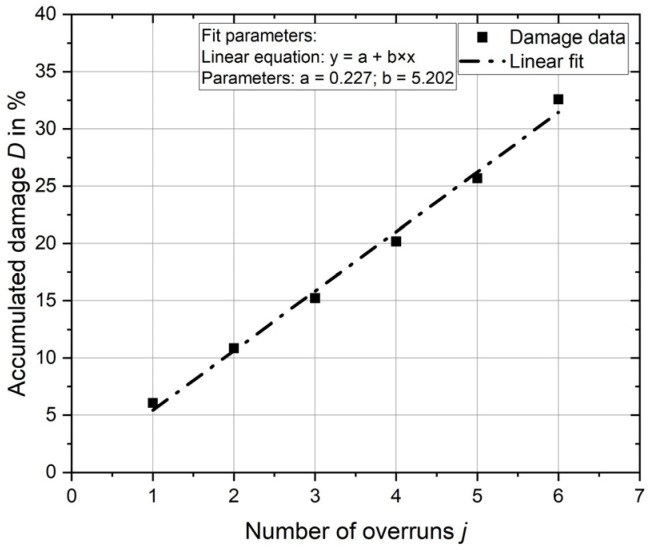
Calculated cumulative damage and linear regression.

In Figure 6, the total damage *D* is plotted over the number of deep rolling overruns *j* for Experiment 1. A linear increase in damage is found for both Experiments, and the total damage is, therefore, linearly approximated. The parameters of the equation are given for Experiment 1 in the diagram. A similar trend is observed for Experiment 2. The parameters for the linear approximation of Experiment 2 are a = 1.261 and b = 2.466.

Using the linear equations, the damage can be extrapolated linearly until the damage sum is greater than 100%. Table 3 shows these results compared with the results of the experiments.

For Experiment 1, damage exceeding *D* = 100% is calculated after 20 deep rolling overruns compared with the experimental result of 23 overruns. The calculated damage sum for 23 deep rolling overruns is 119.9%.

The damage exceeding *D* = 100% for Experiment 2 is calculated after 41 repeated deep rolling overruns. In comparison, the number of overruns in the experiment is 48. The calculated damage sum for 48 deep rolling overruns is 119.6%.

The calculation method presented provides a reliable means of determining the damage caused by the deep rolling process. The calculation gives conservative results for the damage sum *D* = 100%. The almost identical size of the damage sum where damage was found in the experiments of ~120% proves that the presented assessment method works reliably for different deep rolling parameters.

The evaluation method is applied in the same way to evaluation points below the surface. This is to ensure that the worst damage caused by deep rolling does not occur below the surface and that cracking does not start there and then grow to the surface. In both Experiments 1 and 2, the greatest damage is found on the surface after one deep rolling overrun and also after several overruns.

### 3.3. Investigated Parameters and Optimisation

The valid calculation method is used to assess the damage of different rolling scenarios. As described in Section 2.3, the most influential deep rolling parameters, deep rolling force and feed rate, are analysed. In addition, the damage caused by optimising the deep rolling treatment with multiple deep rolling at reduced forces is investigated.

#### 3.3.1. Deep Rolling Force

**Figure 7:**
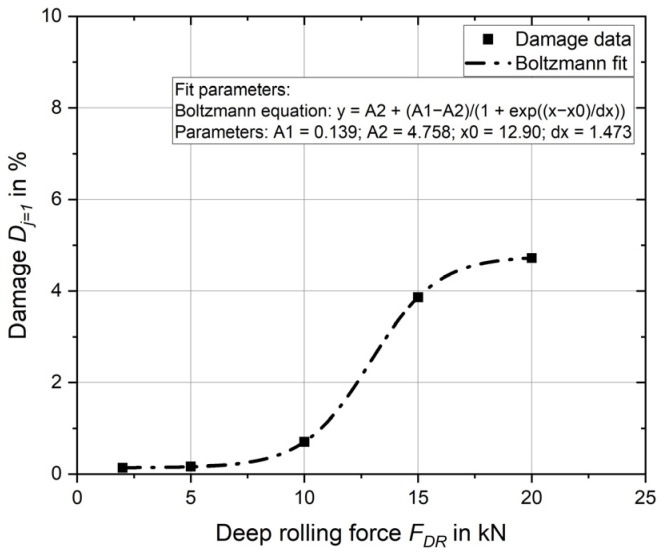
Influence of deep rolling force on damage introduced.

Firstly, the influence of the deep rolling force on the damage introduced is investigated for one overrun (*j* = 1). The deep rolling forces 20 kN, 15 kN, 10 kN, 5 kN and 2 kN are considered and analysed exclusively. The feed rate is kept constant at 0.5 mm. Five separate simulation models are set up and simulated with the appropriate forces. The required strain and stress are then evaluated, and the damage for a deep rolling overrun with the corresponding deep rolling force is determined using the evaluation method presented. The result of the evaluation is shown in Figure 7. It displays the damage with the deep rolling force. The points plotted are the evaluated results. A suitable equation is found to describe the relationship between damage and deep rolling force. The relationship, equation and parameters are shown in the diagram.

For the deep rolling forces 2 kN, 5 kN and 10 kN, the calculated damage for one overrun *D*_*j*=1_ is less than 1%. From 10 kN there is a significant increase in damage up to 15 kN, where 3.9% damage already occurs. The increase flattens out up to 20 kN, where the 4.7% damage already known from the results of Experiment 2 can be seen.

#### 3.3.2. Feed Rate

Subsequent analysis focuses on the influence of the feed rate for one overrun (*j* = 1). Again, the influence of feed rate is explicitly analysed; the deep rolling force is always 20 kN. In addition to the feed rates FR_1_ = 0.25 mm from Experiment 1 and FR_2_ = 0.5 mm from Experiment 2, the feed rates 1.0 mm and 2.0 mm are also considered.

Again, appropriate simulation models are set up and analysed, and the damage calculation is applied. Feed rate has a direct effect on the number of times the same point is machined by the tools, so it is expected that the damage will decrease as feed rate increases.

**Figure 8:**
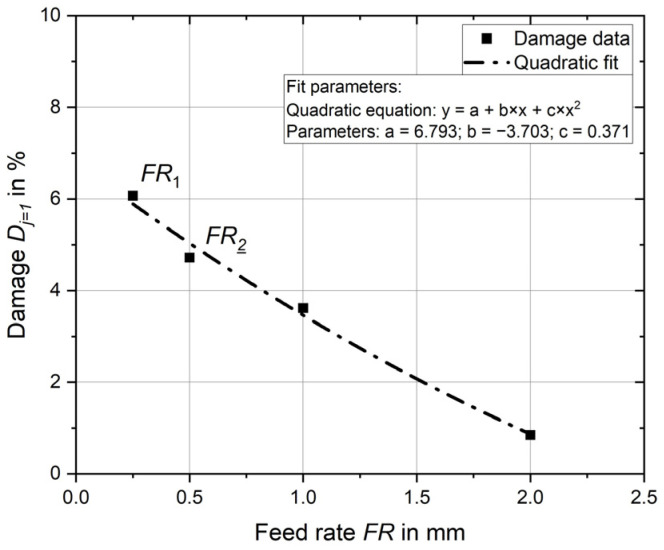
Influence of feed rate on damage introduced.

Exactly this behaviour is shown in Figure 8, where the results of the evaluation are again shown as points and the relationship between damage and feed rate is described by an equation. A quadratic equation is suitable for describing the behaviour here, and the parameters are listed in the figure.

The damage result at a feed rate of 0.25 mm is already known from Experiment 1 to be 6.1%, and at a feed rate of 0.5 mm from Experiment 2 to be 4.7%. The damage continues to decrease as feed rate increases. For a feed rate of 1.0 mm, it is 3.6%, and for a feed rate of 2.0 mm, damage decreases further to 0.8%.

#### 3.3.3. Process Optimisation

Finally, the influence of optimising the deep rolling application presented in [15] on the induced damage is investigated. It was found that repeated deep rolling with reduced deep rolling forces has a positive effect on the induced residual stress state. Furthermore, the fatigue strength assessment based on the residual stress state presented in [17] shows that the optimisation allows a significant increase in load capacity.

The basis for the assessment is the simulation model for deep rolling at 20 kN and a feed rate of 0.5 mm, Experiment 2, with the calculated introduced damage of 4.7%. This result is again used as a reference. Based on this simulation model, three additional models are set up. Firstly, the model that has already been deep rolled at 20 kN is once subjected to an additional overrun at 5 kN (20 kN/5 kN), once to an additional overrun at 10 kN (20 kN/10 kN) and once at 10 kN and, finally, at 5 kN (20 kN/10 kN/5 kN).

Multiple deep rolling is simulated, the simulation models are evaluated, and the damage calculation is performed. Similar to the validation of the calculation method, Section 3.2, the damage from the additional deep rolling overruns is accumulated.

An additional deep rolling overrun of 5 kN increases the damage from 4.7% to 4.8%. If 10 kN is applied in addition to 20 kN, the damage increases to 5.2%, and with triple treatment at 20 kN, 10 kN and 5 kN, damage of 5.3% must be expected.

**Figure 9:**
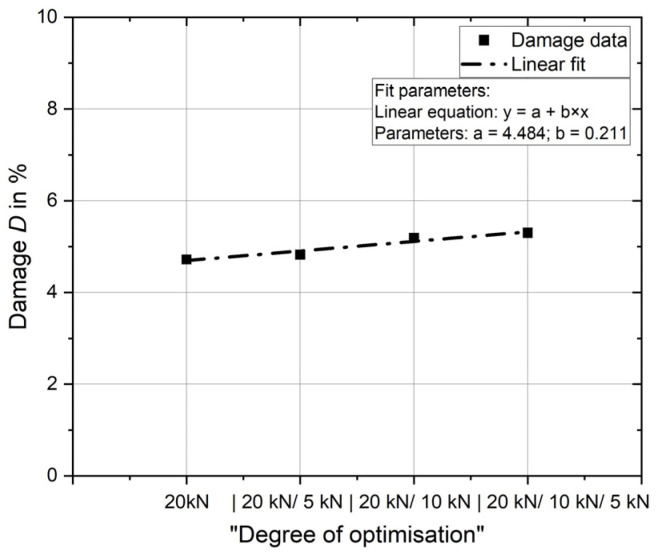
Influence of process optimisation on damage introduced.

The results are shown in Figure 9. The damage is plotted against the increasing “degree of optimisation”. The relationship can be described in simplified terms by using a linear equation. The parameters are shown again in the diagram.

According to [17], triple deep rolling (20 kN/10 kN/5 kN) allows a significant increase in fatigue strength. The increase in damage introduced is small and, due to the reduced forces, it is not expected that the increase in damage will have a negative effect on fatigue strength.

## 4. Conclusions

In this paper, the damage caused by deep rolling is investigated experimentally and a valid assessment method is presented. In addition, the main deep rolling parameters and the optimisation by repeated deep rolling with reduced deep rolling forces are investigated. The main results are summarised in the following list:

- A railway axle is repeatedly deep rolled on two cylindrical sections until damage is detected. A feed rate of 0.25 mm is used for Experiment 1 and a feed rate of 0.5 mm for Experiment 2. In Experiment 1, damage occurs after 23 deep rolling overruns, and in Experiment 2, after 48 deep rolling overruns.
- To quantify the damage caused by deep rolling, an appropriate damage assessment method is developed. The comparison of the number of deep rolling overruns from the experiment and the calculation with occurring damage or an exceeding damage sum of 100% for Experiment 1 is 20 and 23 deep rolling overruns, respectively, and 41 and 48 deep rolling overruns, respectively, for Experiment 2.
- The calculated damage sum for the number of overruns where damage was found in the experiments is around 120% for both experiments. The calculation shows strong applicability for different deep rolling parameters and provides conservative results.
- The developed assessment is applied to the parameters of deep rolling force and feed rate for one deep rolling overrun. The highest damage is introduced with the highest deep rolling force of 20 kN at 4.7% and the lowest feed rate of 0.25 mm at 6.1%.
- Finally, the optimisation of deep rolling with repeated deep rolling at reduced forces is analysed. With three overruns at 20 kN, followed by 10 kN and finally 5 kN, the damage increases just by 0.6 percentage points from 4.7% to 5.3% compared with a single overrun at 20 kN.

This paper presents a calculation method to compare the damage caused by different deep rolling parameters. Of particular interest is the effect and optimum “damage” applied to fatigue strength. Therefore, fatigue tests on railway axles with different applied damages are planned. In this way, the permissible “pre-damage” caused by the application of the deep rolling process can be determined without any negative effect on fatigue strength. Values well below 1 or 100% are assumed.

## Figures and Tables

**Table 1:** Deep rolling parameters.

Deep Rolling Parameters	Symbol	Unit	Value
Deep rolling force	*F_DR_*	kN	20
Deep rolling disc tool geometry			
Diameter	*D*	mm	100
Tip radius	*TR*	mm	9
Feed rate distance			
Experiment 1	*FR*_1_	mm	0.25
Experiment 2	*FR*_2_	mm	0.5

**Table 2:** Parameters of the Uniform Material Law (UML) [14,35,36].

Parameter	Symbol	Formula	Unit	Value
Young’s modulus	*E*	-	MPa	217,836
Ultimate tensile strength	*R_m_*	-	MPa	1015
Fatigue strength coefficient	${\sigma \prime }_{f}$	$=1.5\times R_{m}$	MPa	1522.5
Fatigue strength exponent	*b*	=−0.087	-	−0.087
Fatigue ductility coefficient	${\varepsilon \prime }_{f}$	$=0.59\times (1.375-125\times R_{m}/E)$	-	0.4676
Fatigue ductility exponent	*c*	=−0.58	-	−0.58

**Table 3:** Comparison between experimental and calculated results.

	Experiment 1	Experiment 2
**Experimental**		
Number of overruns when damage is found	23	48
**Calculation**		
Number of overruns exceeding *D* = 100%	20	41
Calculated total damage at number of overruns when experimental damage is found	*D*_*j*=23_ = 119.9%	*D*_*j*=48_ = 119.6%

## Data Availability

Data are contained within the article.

## References

[1] Delgado P., Cuesta I.I., Alegre J.M., Díaz A. (2016). State of the art of Deep Rolling. Precis. Eng..

[2] Altenberger I. (2005). Deep Rolling—The Past, the Present and the Future. Altern. Process..

[3] Schulze V. (2006). Modern Mechanical Surface Treatment: States, Stability, Effects.

[4] Scholtes B., Voehringer O. (2001). Mechanical surface treatment. Encyclopedia of Materials: Science and Technology.

[5] Bäcker V., Klocke F., Wegner H., Timmer A., Grzhibovskis R., Rjasanow S. (2010). Analysis of the deep rolling process on turbine blades using the FEM/BEM-coupling. IOP Conf. Ser. Mater. Sci. Eng..

[6] Fischer A., Scholtes B., Niendorf T. (2021). Influence of Deep Rolling and Induction Hardening on Microstructure Evolution of Crankshaft Sections made from 38MnSiVS5 and 42CrMo4. HTM J. Heat Treat. Mater..

[7] Regazzi D., Beretta S., Carboni M. (2014). An investigation about the influence of deep rolling on fatigue crack growth in railway axles made of a medium strength steel. Eng. Fract. Mech..

[8] Marschnig S. (2016). Innovative Track Access Charges. Transp. Res. Procedia.

[9] Verkehr B.F. (2022). 3003 Bern, Abteilung Finanzierung, “Basispreis Verschleiss im Trassenpreis: Anleitung für die Fahrzeugpreisbestimmung”. https://www.bav.admin.ch/dam/bav/de/dokumente/verordnungen_desdepartements/eisenbahn/nzv-bav-anleitung.pdf.download.pdf/Anleitung_DE_Verschleissfaktor_Fahrbahn.pdf.

[10] (2022). Railway Applications—Wheelsets and Bogies—Part 1: Design Method for Axles with External Journals.

[11] (2020). Railway Applications—Wheelsets and Bogies—Axles—Product Requirements.

[12] Röttger K., Wilcke G., Mader S. (2005). Festwalzen—eine Technologie für effizienten Leichtbau. (in Deu). Mat.-Wiss. Werkst..

[13] Radaj D., Vormwald M. (2003). Ermüdungsfestigkeit: Grundlagen für Leichtbau, Maschinen- und Stahlbau.

[14] Pertoll T., Buzzi C., Dutzler A., Leitner M., Seisenbacher B., Winter G. (2023). Experimental and numerical investigation of the deep rolling process focussing on 34CrNiMo6 railway axles. Int. J. Mater. Form..

[15] Pertoll T., Buzzi C., Leitner M., Boronkai L. (2024). Numerical parameter sensitivity analysis of residual stresses induced by deep rolling for a 34CrNiMo6 steel railway axle. Int. J. Adv. Manuf. Technol..

[16] Pertoll T., Buzzi C., Leitner M., Simunek D., Boronkai L. (2024). Residual life assessment of deep rolled railway axles considering the effect of process parameters. Procedia Struct. Integr..

[17] Pertoll T., Buzzi C., Leitner M., Boronkai L. Application of local fatigue strength approach to assess and optimise the impact of deep rolling on the fatigue performance of railway axles. Int. J. Fatigue.

[18] Callister W.D., Rethwisch D.G. (2018). Materials Science and Engineering: An Introduction.

[19] Mouritz A.P. (2012). Introduction to Aerospace Materials: From Physical Principles to Turnkey Instrumentation.

[20] Kuna M. (2008). Numerische Beanspruchungsanalsye von Rissen: Finite Elemente in der Bruchmechanik; mit Zahlreichen Beispielen.

[21] Hull R., Messerschmidt U. (2010). Dislocation Dynamics during Plastic Deformation.

[22] Antunes R.A., de Oliveira M. (2015). Effect of surface treatments on the fatigue life of magnesium and its alloys for biomedical applications. Surface Modification of Magnesium and Its Alloys for Biomedical Applications.

[23] Starker P., Macherauch E. (1983). Kugelstrahlen und Schwingfestigkeit. Mater. Werkst.

[24] Cammett J. (2014). Are You Peening Too Much?. Shot Peen..

[25] Prevey P.S., Cammett J.T. (2005). The Effect of Shot Peening Coverage on Residual Stress, Cold Work and Fatigue in a Ni-Cr-Mo Low Alloy Steel. Proc. Int. Conf. Shot Peen..

[26] Llaneza V., Belzunce F.J. (2015). Optimal Shot Peening Treatments to Maximize the Fatigue Life of Quenched and Tempered Steels. J. Mater. Eng. Perform..

[27] Liu W., Dong J., Zhang P., Zhai C., Ding W. (2009). Effect of Shot Peening on Surface Characteristics and Fatigue Properties of T5-Treated ZK60 Alloy. Mater. Trans..

[28] Champaigne J. (1992). Shot Peening Intensity Measurement. Shot Peen..

[29] GmbH O.S. Definition of Shot Peening Control and Parameters. https://osk-kiefer.de/wp-content/uploads/28-definition_of_shot_peening_control_and_parameters_go.pdf.

[30] Kirk D. (2016). Peening Intensity: True Meaning and Measurement Strategy. Shot Peen..

[31] Berstein G., Fuchsbauer B. (1982). Festwalzen und Schwingfestigkeit. (in Deu). Z. Werkst..

[32] Dänekas C., Heikebrügge S., Schubnell J., Schaumann P., Breidenstein B., Bergmann B. (2022). Influence of deep rolling on surface layer condition and fatigue life of steel welded joints. Int. J. Fatigue.

[33] Kloos K.H., Adelmann J. (1988). Schwingfestigkeitssteigerung durch Festwalzen. Mat.-Wiss. Werkstofftech..

[34] Regazzi D., Cantini S., Cervello S., Foletti S., Pourheidar A., Beretta S. (2020). Improving fatigue resistance of railway axles by cold rolling: Process optimisation and new experimental evidences. Int. J. Fatigue.

[35] Bäumel A., Seeger T. (1990). Materials Data for Cyclic Loading: Supplement 1.

[36] Haibach E. (2006). Betriebsfestigkeit: Verfahren und Daten zur Bauteilberechnung.

[37] Smith K., Watson P., Topper T. (1970). A stress-strain function for the fatigue of metals. J. Mater..

[38] Miner M.A. (1945). Cumulative Damage in Fatigue. J. Appl. Mech..

